# Damage Accrual in Patients with Systemic Lupus Erythematosus Predicts Mortality and Is Associated Primarily with Antiphospholipid Syndrome and Hypertension

**DOI:** 10.3390/jcm15103667

**Published:** 2026-05-10

**Authors:** Yael Pri-Paz Basson, Hadar Haim-Pinhas, Daniel Erez, Iftach Sagy, Keren Cohen-Hagai, Shaye Kivity, Oshrat E. Tayer-Shifman

**Affiliations:** 1 Rheumatology Unit, Meir Medical Center, Kfar Saba 4428164, Israel; 2 Gray Faculty of Medical and Health Sciences, Tel Aviv University, Tel Aviv 6997801, Israel; 3 Department of Nephrology and Hypertension, Meir Medical Center, Kfar Saba 4428164, Israel; 4 Internal Medicine D, Meir Medical Center, Kfar Saba 4428164, Israel; 5 Rheumatic Disease Unit, Soroka University Medical Center, Beer-Sheva 84101, Israel; 6 Faculty of Health Sciences, Ben Gurion University of the Negev, Beer-Sheva 84105, Israel; 7 Clinical Research Center, Soroka University Medical Center, Beer Sheva 84101, Israel

**Keywords:** systemic lupus erythematosus, damage accrual, antiphospholipid syndrome, hypertension, mortality

## Abstract

**Background/Objectives:** Long-term outcomes in systemic lupus erythematosus (SLE) are largely driven by irreversible organ damage, yet the relative contribution of comorbid conditions remains insufficiently characterized. We aimed to characterize damage accrual and identify comorbidities associated with damage severity and mortality. **Methods:** A retrospective study of adult patients with SLE followed at a single-center (2014–2023). The Systemic Lupus International Collaborating Clinics/American College of Rheumatology Damage Index (SDI), was used to assess damage at last follow-up. Damage was categorized as none (0), mild–moderate (1–2), or severe (≥3). Demographic, clinical, laboratory, treatment, and comorbidity data were extracted from electronic medical records. Multivariable logistic regression and Cox proportional hazards models were applied to identify factors associated with damage severity and mortality. **Results:** Among 182 patients (84.1% female; mean follow-up 15.6 ± 11.4 years), 59.5% accrued damage, including 30.8% with severe damage. Damage predominantly involved cardiovascular, ocular, neuropsychiatric, and musculoskeletal domains. It was associated with older age, longer disease duration, hematologic and renal involvement, and corticosteroids and immunosuppressive medications. In multivariable analysis, antiphospholipid syndrome (APS) and hypertension emerged as the dominant independent predictors of damage accrual with an odds ratio of 15.70 (95% CI 4.26–57.89, *p* < 0.001) and 6.46 (95% CI 2.54–16.40, *p* < 0.001), respectively. Mortality increased with damage severity (16.1% in SDI ≥ 3, 1.9% in SDI 1–2, none in SDI = 0; *p* < 0.0001). Damage was also associated with increased hospitalizations. **Conclusions:** Damage accrual is common and strongly predicts mortality. APS and hypertension emerge as dominant, modifiable drivers, supporting integrated cardiovascular and thrombotic risk management in SLE.

## 1. Introduction

Systemic lupus erythematosus (SLE) is a complex chronic autoimmune disease characterized by marked clinical heterogeneity and involvement of multiple organ systems [1]. Despite advances in diagnosis and management, patients with SLE continue to have a mortality rate approximately twice that of the general population, with only modest improvement over recent decades [2]. One of the principal contributors to excess mortality and long-term morbidity in SLE is the accrual of organ damage over time. Patients with higher levels of damage are at a greater risk of premature death [3,4].

Irreversible organ damage in SLE is commonly assessed using the Systemic Lupus International Collaborating Clinics/American College of Rheumatology Damage Index (SDI). This validated instrument captures irreversible damage occurring after the diagnosis of SLE, irrespective of attribution [4,5]. Damage accrual has consistently been shown to predict adverse outcomes, including hospitalization, impaired quality of life, and premature mortality [1,2,3,6]. Given the prognostic significance of damage accrual, current recommendations for the management of SLE emphasize damage prevention as a central therapeutic goal and stress the importance of assessing damage regularly [7,8,9].

Damage accrual in SLE reflects the interplay between disease activity, treatment exposure (particularly glucocorticoids), antiphospholipid antibodies, genetic susceptibility, demographic characteristics, and comorbid conditions such as antiphospholipid syndrome (APS), hypertension, diabetes, and dyslipidemia [1,4,5,10,11,12]. Importantly, both the burden and pattern of damage vary across populations and healthcare settings, influenced by ethnicity, socioeconomic factors, disease phenotype, and access to care [4,5,13,14]. In recent years, there has been growing recognition that long-term outcomes in SLE should be approached within a broader, outcome-oriented framework that extends beyond disease activity alone [1,7]. Treat-to-target strategies, focused initially on achieving remission or low disease activity, are increasingly incorporating additional goals such as prevention of irreversible damage, minimization of treatment toxicity, and improvement in patient-reported outcomes [7,9,15,16,17]. Within this evolving paradigm, comorbid conditions have emerged as critical and potentially modifiable contributors to damage accrual, in some cases acting independently of inflammatory disease activity [4,10,18,19]. Despite these advances, there remains a relative paucity of data from well-characterized regional cohorts, particularly in Middle Eastern populations, where demographic and healthcare system differences may influence long-term outcomes. Addressing this gap is essential to refining risk stratification and tailoring management strategies to specific patient populations.

The present study aimed to characterize long-term damage accrual in a contemporary Israeli SLE cohort and to identify factors associated with damage severity and mortality, with a focus on the contribution of comorbid conditions.

## 2. Materials and Methods

### 2.1. Study Design and Population

This retrospective, single-center cohort study was conducted at Meir Medical Center (MMC), an academic hospital in central Israel providing both inpatient and outpatient care to a diverse urban population. The study utilized data extracted from the institution’s electronic medical records (EMR), which include longitudinal clinical, laboratory, and administrative data across all healthcare encounters. Electronic medical records were available from 2014 onward; however, historical clinical data, including year of diagnosis and prior disease manifestations, were extracted from physician-documented medical history within the records to capture the longitudinal disease course. Data prior to 2014 may therefore be subject to incomplete ascertainment.

Eligible participants were adults (≥18 years) with a confirmed diagnosis of SLE who were followed at MMC between 1 January 2014, and 31 December 2023. Inclusion required fulfillment of the 2019 EULAR/ACR classification criteria for SLE [20] and a minimum disease duration of 12 months to allow for meaningful assessment of damage accrual. All diagnoses were verified through a detailed chart review by an experienced rheumatologist (OETS). Patients with incomplete clinical data precluding assessment of damage status were excluded; patients with malignancies or other significant comorbidities were not excluded.

The study was approved by the Institutional Review Board of Meir Medical Center (approval number: 0041-22-MMC; date: 15 June 2022). A full waiver of informed consent was granted by the IRB, in accordance with local regulations, as this is a retrospective study based on anonymized data extracted from patients’ medical records.

### 2.2. Outcomes and Definitions

The primary outcome was irreversible organ damage, assessed using the SDI at the last available follow-up. Patients were classified into three categories according to the damage severity based on SDI scores: no damage (SDI = 0), mild-to-moderate damage (SDI 1–2), and severe damage (SDI ≥ 3), to reflect increasing levels of damage burden. This approach is consistent with prior studies that have stratified damage into ordinal categories [4,6,13], including the use of SDI ≥ 3 thresholds to represent more severe damage [21].

Sociodemographic variables included age, sex, ethnicity, and socioeconomic status, the latter determined using the national municipal socioeconomic index. Cumulative SLE manifestations were defined based on the clinical domains included in the 2019 EULAR/ACR classification criteria and were recorded as present if documented at any time during the disease course. Disease-related variables included organ involvement, laboratory parameters (including complement levels, anti-dsDNA antibodies, hematologic indices, and renal function), and treatment exposures such as glucocorticoids, hydroxychloroquine, immunosuppressive agents, and biologic therapies. Treatment exposure and laboratory abnormalities were defined as "ever" if documented at any time during the disease course. Glucocorticoid exposure was additionally assessed as mean daily dose at the last follow-up. For laboratory parameters, the minimum recorded value during the available follow-up period was used for analysis.

Comorbidities were defined based on physician-documented diagnoses in the EMR. Hypertension was defined as a documented diagnosis and/or use of antihypertensive medications. Diabetes mellitus and end-stage renal disease (ESRD) were defined based on documented clinical diagnoses. APS was defined according to the revised Sapporo APS classification criteria [22], requiring thrombotic and/or obstetric manifestations in the presence of persistent antiphospholipid antibodies. Cardiovascular disease included ischemic heart disease, stroke, and heart failure. Healthcare utilization outcomes included emergency department visits and hospitalizations, categorized according to the primary documented diagnosis in the medical record as disease flare, infection, or cardiovascular event. All-cause mortality data were obtained from national registries and included both in-hospital and out-of-hospital deaths.

### 2.3. Statistical Analysis

Continuous variables are presented as mean ± standard deviation (SD), and categorical variables as counts and percentages. Group comparisons were performed using Student’s *t*-test for continuous variables and chi-square tests for categorical variables, as appropriate. When overall group differences were statistically significant, post hoc pairwise comparisons were performed using Mann–Whitney U test for continuous variables and chi-square or Fisher’s exact tests for categorical variables, with Bonferroni correction applied for multiple comparisons (adjusted significance threshold *p* < 0.017).

To identify independent predictors of damage accrual, multivariable logistic regression models were constructed. Variables included in the multivariable models were selected based on clinical relevance and significance in the univariable analyses. A multivariable Cox proportional hazards model was used to evaluate factors associated with mortality, adjusting for age and sex. Analyses were conducted using available data.

All analyses were conducted using SPSS software (version 29; IBM Corp., Armonk, NY, USA). A two-sided *p*-value < 0.05 was considered statistically significant.

## 3. Results

### 3.1. Patients’ Characteristics

A total of 182 patients were followed for a mean duration of 15.6 ± 11.4 years. Of these, 84.1% were female, 74.7% were Jewish, and 22.5% were Arab. Socioeconomic status was categorized as high in 48.1% of patients and low in 15.5% (Table 1). Hydroxychloroquine was used at some point during the disease course in 98.3% of patients; data regarding its use were missing for 1.65% of patients. The most prevalent comorbidities at the end of follow-up were hypertension (32.4%) and APS (23.6%). Additionally, 9.9% of patients had diabetes mellitus (DM), and 10.4% had cardiovascular disease (CVD) (Table 1). Cumulative SLE manifestations in the cohort were dominated by joint involvement (147 patients, 80.8%) and cutaneous manifestations (99, 54.4%). Hematologic involvement was also common, affecting 81 patients (44.5%), followed by renal involvement in 70 (38.5%). Serositis was observed in 38 patients (20.9%), while fever (21, 11.5%) and neuropsychiatric involvement (10, 5.5%) were less frequent. Among the 70 patients with renal manifestations, kidney biopsy data were available for 45 patients. Class IV ± class V was the most common pattern (23 patients, 51.1%), followed by class III ± class V (12, 26.7%). Pure class V disease was observed in 9 patients (20.0%), and class II in 1 (2.2%).

### 3.2. Prevalence of Damage Accrual

**Figure 1:**
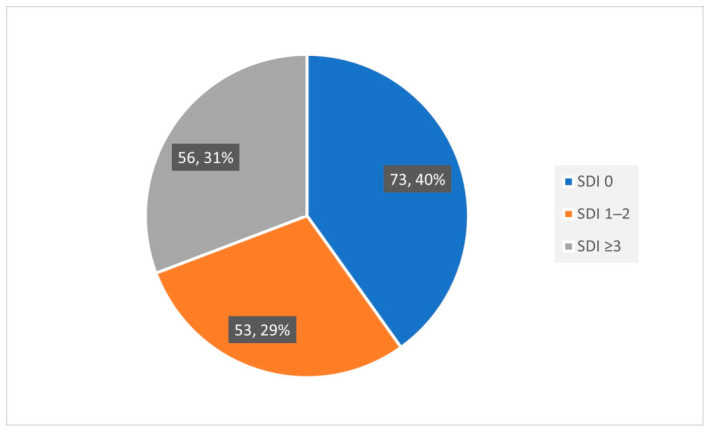
Distribution of damage severity according to the Systemic Lupus International Collaborating Clinics/American College of Rheumatology Damage Index (SDI) among patients with systemic lupus erythematosus. SDI 0 indicates no damage, SDI 1-2 indicates mild-to-moderate damage, and SDI ≥3 indicates severe damage.

Damage had occurred in 59.5% of the cohort (109 patients). Of them, 53 (48.6%) had mild to moderate damage, and 56 (51.4%) had severe damage (Figure 1). The mean SDI score was 1.88 ± 2.34 and the median (IQR) score was 1 (0–3).

**Figure 2:**
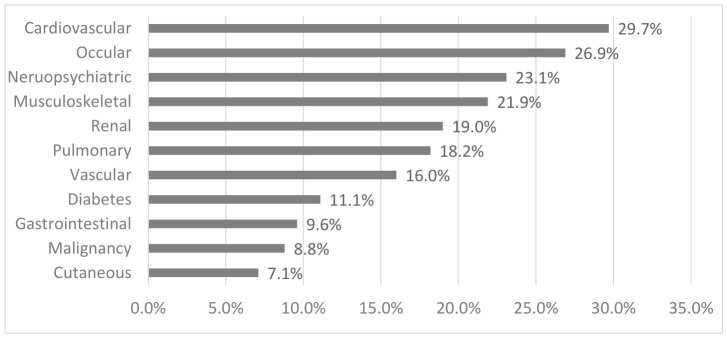
Distribution of organ-specific damage domains according to the Systemic Lupus International Collaborating Clinics/American College of Rheumatology Damage Index (SDI) among patients with systemic lupus erythematosus. Percentages represent the proportion of patients with damage in each organ domain.

The most commonly affected SDI domains were cardiovascular (29.7%), ocular (26.9%), neuropsychiatric (23.1%), and musculoskeletal (21.9%) (Figure 2). Within the cardiovascular domain, valvular disease was the most common manifestation, affecting 23 patients (40.4%), followed by myocardial infarction in 15 patients (26.3%) and ventricular dysfunction in 12 (21.1%). Within the ocular domain, cataract was the predominant finding, present in 40 patients (71.4%), while retinal atrophy/retinal damage was observed in 16 patients (28.6%). Within the neuropsychiatric domain, stroke was the most frequent, occurring in 17 patients (37.8%), followed by cognitive impairment/major psychosis in 16 (35.6%), and seizures in 7 (15.6%). Within the musculoskeletal domain, osteoporosis with fracture/vertebral collapse was the most common manifestation, affecting 17 patients (56%), followed by osteomyelitis in 6 (20%) and deforming/erosive arthritis in 4 (13.3%).

### 3.3. Factors Associated with Damage Accrual

Patients with damage were older, had a longer follow-up, had higher cumulative EULAR/ACR criteria scores, and exhibited more frequent hematologic and renal involvement. These factors were associated not only with damage accrual but also with its severity (Table 1). Comorbidities, including hypertension, diabetes, cardiovascular disease, end-stage renal disease, and APS were significantly associated with damage accrual. In contrast, antiphospholipid antibodies (aPL) positivity alone was not significantly associated with damage accrual. Damage accrual was also associated with lower hemoglobin levels, lymphopenia, and thrombocytopenia (Table 1).

The use of corticosteroids and immunosuppressives at any time was associated with damage accrual, as well as with mean glucocorticoid dosage at last follow-up, but no association was found with the use of belimumab (Table 1).

Although the severe damage group included a larger proportion of Arab patients and those from a lower socioeconomic status, neither socioeconomic status nor ethnicity (Jewish/Arab) was statistically significant (Table 1).

Post hoc pairwise comparisons using Mann–Whitney U test for continuous variables and chi-square or Fisher’s exact tests for categorical variables, with Bonferroni correction, demonstrated that most significant differences were driven by the severe damage group (SDI ≥ 3), which differed consistently from both the no-damage and mild-to-moderate groups. In contrast, differences between the no-damage and mild-to-moderate groups were generally not statistically significant. Hypertension demonstrated a stepwise increase across damage categories, with significant differences between groups, indicating a true gradient association with damage accrual. APS was significantly higher in both the mild-to-moderate and severe groups compared to patients without damage, but did not differ significantly between the mild-to-moderate and severe groups, suggesting an association with damage presence rather than progressive severity.

### 3.4. Multivariable Analysis and Outcomes

In the multivariable regression analysis model, APS and hypertension emerged as the strongest independent predictors of damage accrual (Table 2).

**Figure 3:**
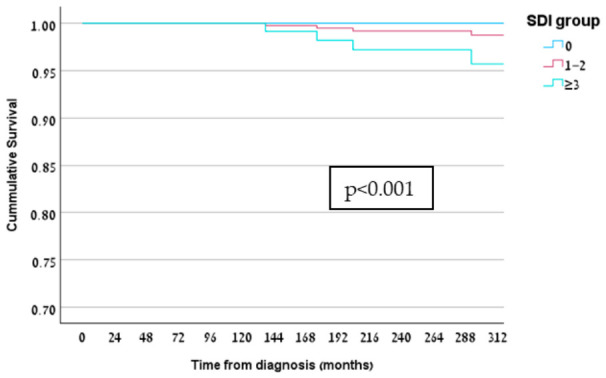
Age- and sex-adjusted Cox survival model according to SDI group. SDI: Systemic Lupus International Collaborating Clinics/American College of Rheumatology Damage Index.

Mortality occurred in 16.1% of patients with SDI ≥ 3, compared with 1.9% in the SDI 1–2 group and no deaths in patients without damage (*p* < 0.0001) (Table 1, Figure 3). Damage was also associated with hospitalizations for severe infections, SLE flares, and emergency department visits (Table 1).

## 4. Discussion

In this long-term, single-center Israeli cohort, irreversible organ damage was observed in nearly 60% of patients with SLE after a mean follow-up of over 15 years. Damage accrual was strongly associated with adverse clinical outcomes, including hospitalization and mortality, reinforcing the central role of damage prevention in contemporary SLE management. Multivariable analysis demonstrated that APS and hypertension were the strongest independent predictors of damage accrual.

The prevalence and severity of damage observed in our cohort are within the range reported in previous reports from Israel and international cohorts. A prior study in Israel by Molad et al. [23] reported a comparable damage rate of 61.6%, with a mean SDI of 1.64 ± 2.1, despite a considerably shorter mean follow-up of 3.8 ± 3.12 years. Since damage accrual in SLE is known to increase progressively over time [3], the similarity in mean SDI scores between our longer-term study and Molad’s earlier cohort are notable and may reflect differences in cohort characteristics, management strategies, or disease severity. However, our findings align with global data. A prior study showed that 20% of SLE patients acquire damage during the first year after diagnosis with a decline in annual rate of new damage to 5% beyond year 5 [24]. The SLICC Inception Cohort [4] reported that 51.1% of patients had accrued at least one item of damage within six years of clinic entry. Likewise, studies from Brazil [25] and China [13] reported damage accrual rates of 55% and 42.8%, respectively. Collectively, these observations underscore the substantial global burden of irreversible organ damage in SLE and highlight the SDI as a reliable, cross-contextual measure for long-term outcomes in diverse patient populations.

We found that the use of corticosteroids and immunosuppressive agents at any time was associated with damage accrual, as was mean glucocorticoid dose at last follow-up, whereas no such association was observed for belimumab. Glucocorticoids are well established as a major contributor to irreversible organ damage in SLE [5,7]. Accordingly, current treatment strategies emphasize early use of steroid-sparing agents, including biologic therapies, to minimize cumulative glucocorticoid exposure and its associated damage [1,7,9]. Several studies have suggested that earlier introduction of biologic therapy may contribute to reduced damage accrual [1,26,27]. However, our cohort spans a prolonged period that predates the widespread clinical use of biologics and the implementation of contemporary treat-to-target strategies which may have influenced both the prevalence and severity of damage in our cohort, as evolving treatment strategies over time likely impacted outcomes. This likely explains the lack of an observed protective effect of belimumab in our study, as a substantial proportion of patients were managed under earlier treatment paradigms, and belimumab was often introduced later in the disease course.

The pattern of organ involvement, predominantly cardiovascular, ocular, neuropsychiatric, and musculoskeletal, mirrors findings from the SLICC inception cohort, the Spanish Society of Rheumatology Lupus Registry and earlier Israeli studies [3,23,24,25,28]. There were higher rates of renal damage in some cohorts, mostly in non-Caucasian populations [13,25], that may reflect ethnic differences in disease phenotype and severity. Notably, we observed a higher prevalence of musculoskeletal damage than in the earlier Israeli cohort by Molad et al. [23], which aligns with Gladman et al.’s observation that musculoskeletal damage frequently emerges as a late complication, typically after five years of disease [3]. Overall, our findings support a shared profile of organ damage in SLE, largely independent of geographic or ethnic background, while also highlighting some potential variations in domain-specific damage prevalence.

This study extends the current framework by demonstrating the substantial contribution of comorbid conditions to overall damage burden. In particular, APS and hypertension emerged as key contributors to long-term organ damage in SLE. While disease activity and corticosteroid treatment have traditionally been viewed as major drivers of damage accrual [17,29,30], our findings suggest that comorbidity-related mechanisms play an independent and clinically meaningful role, supporting a shift toward a more integrated model that incorporates both immune-mediated and non-immune determinants of long-term outcomes.

APS emerged as the strongest independent predictor of damage accrual, conferring a markedly increased risk of severe irreversible organ damage. Nearly half of patients with severe damage had concomitant APS, compared with 4% of patients with no damage, underscoring the profound impact of thrombo-inflammatory mechanisms on long-term outcomes in SLE. Notably, in our study, aPL positivity alone was not significantly associated with damage accrual. Prior studies on the association between aPL or APS and damage accrual in patients with SLE reported heterogeneous and sometimes inconsistent results; however, multiple studies support the association between APS and increased damage in SLE [11,31,32,33]. In a Spanish multicenter cohort, SLE patients with APS had significantly higher SLICC damage scores and mortality compared with both aPL-positive patients without APS and those without aPL [11]. SLE-APS patients also demonstrate higher prevalence of neuropsychiatric, cardiac, pulmonary, renal, and ocular manifestations, along with increased cardiovascular risk factors [11,31]. The pattern of damage accrual differs between primary APS and SLE-associated APS. In a longitudinal study [34], SLE-APS patients demonstrated progressive and sustained damage accumulation over time, whereas damage in primary APS occurred predominantly early and showed limited progression during follow-up. Overall, SLE-APS patients accumulated more extensive and progressive damage over time and had higher mortality rates, with effects that appear to exceed those seen in patients with primary APS, SLE without APS, or SLE with isolated aPL positivity without APS, suggesting a synergistic burden in the context of coexisting SLE [11,34]. The high odds observed in our cohort further emphasize the need for vigilant screening, early recognition, and aggressive intervention in patients with APS, particularly those with concurrent hypertension or multiorgan involvement. Integrating APS management into routine SLE care may be essential to reducing irreversible damage and improving long-term outcomes.

Hypertension was the second-strongest independent predictor of damage accrual in our cohort and represents a major contributor to long-term morbidity in SLE. Hypertension is highly prevalent among patients with SLE and occurs more frequently than in the general population [4,35], where it contributes to both cardiovascular disease and renal damage, two leading drivers of long-term morbidity in SLE [35]. Hypertension has been consistently identified as a significant and potentially modifiable risk factor for organ damage accrual [4,12,35,36]. This is supported by our post hoc analyses, which demonstrated a stepwise increase in hypertension prevalence across damage categories, consistent with a gradient relationship between hypertension and damage severity. Beyond its role as a traditional cardiovascular risk factor, hypertension in SLE likely reflects a complex interplay between chronic inflammation, endothelial dysfunction, renal involvement, and treatment-related effects, particularly glucocorticoid exposure [35,37,38]. Several longitudinal cohorts have identified hypertension as an independent predictor of damage accrual. In the Hopkins Lupus Cohort, hypertension was among the key predictors of damage progression alongside disease activity, corticosteroid use, and older age [5]. Similarly, in the SLICC inception cohort, hypertension was associated both with the transition from no damage to damage and with progression of existing damage [4]. Additional studies have linked hypertension to increased risk of stroke, cognitive dysfunction, and renal damage in SLE [12,35,36]. Together, these findings support a central role for hypertension as both a marker and mediator of cumulative organ damage [12,35,36]. The strong association observed in our cohort reinforces the importance of early identification and strict blood pressure control as an integral component of SLE management, particularly in patients with additional risk factors or established organ involvement.

From a clinical perspective, while prior studies have emphasized disease activity and glucocorticoid exposure as the main drivers of damage accrual [15,16,17], our results, as others [1,18], highlight the critical role of comorbidity control, particularly hypertension and APS, in reducing irreversible organ damage. Notably, higher disease activity may itself contribute to the development of comorbidities, particularly cardiovascular and metabolic complications, suggesting a bidirectional relationship between inflammation, comorbidity burden, and long-term outcomes [39]. This concept is further supported by our previous work demonstrating the impact of comorbid conditions on chronic kidney disease progression in SLE [40]. Integrating systematic comorbidity management into routine practice may therefore represent a key opportunity to improve long-term outcomes.

Contrary to previous reports, socioeconomic status and ethnicity were not significantly associated with damage accrual in our cohort, although there was a numerical difference. This finding contrasts with previous studies [5,41], including a study from Israel by Sagy et al. [42], which have demonstrated that low socioeconomic status is significantly associated with poorer outcomes in SLE patients, even within healthcare systems that provide universal coverage. This finding may reflect the centralized location of our medical center, together with Israel’s universal healthcare system, which may alleviate disparities in access to specialized care and advanced therapies.

The strong association between damage severity and mortality observed in this study further confirms the prognostic significance of the SDI [43]. Importantly, no deaths occurred among patients without damage, emphasizing damage accrual as a critical inflection point in long-term SLE outcomes. Prior cohort and meta-analyses have consistently shown that irreversible organ damage independently predicts mortality in SLE, with each 1-point increase in SDI associated with a pooled HR of 1.34 risk of death [6]. Damage involving renal, cardiovascular, neuropsychiatric, and musculoskeletal systems has been particularly linked to increased mortality [44]. These findings underscore the importance of preventing damage accrual through optimal disease control and early targeted intervention to improve long-term survival.

This study has limitations inherent to its retrospective, single-center design, including incomplete data on disease activity and treatment exposure over time. Additionally, while the study includes a diverse population in Israel, the findings cannot be generalized to other populations. Furthermore, our study did not assess mechanistic biomarkers, which could further elucidate the pathophysiology linking clinical factors like hypertension to damage accrual. Nevertheless, its strengths include a long follow-up period, comprehensive clinical data on SLE damage accrual, and inclusion of a diverse Israeli population adding valuable insight into long-term disease management.

## 5. Conclusions

Damage accrual remains a major determinant of morbidity and mortality in SLE. Comorbid conditions, particularly APS and hypertension, are major contributors to irreversible organ damage in SLE and should be considered central targets in long-term disease management. Future research should focus on prospective identification of high-risk patients and on evaluation of targeted interventions to address modifiable comorbidities. Integrating comorbidity management into treat-to-target strategies and assessing its impact on survival and long-term outcomes represent important next steps toward reducing irreversible organ damage and improving long-term outcomes in SLE.

## Figures and Tables

**Table 1:** Demographic and clinical characteristics of patients according to the severity of damage.

	No Damage (SDI 0) *N* = 73	Mild–Moderate Damage (SDI 1–2) *N* = 53	Severe Damage (SDI ≥ 3) *N* = 56	Total *N* = 182	*p*-Value
Female sex, *n* (%)	64 (87.7%)	43 (81.1%)	46 (82.1%)	153 (84.1%)	NS
Age at last follow-up, years, mean ± SD	39.85 ± 13.03	48.89 ± 14.96	59.18 ± 16.02	48.43 ± 16.59	<0.0001
Age at diagnosis, years, mean ± SD	31.12 ± 14.30	34.86 ± 12.69	34.70 ± 16.00	33.3 ± 14.40	NS
Ethnicity, *n* (%)					NS
Jewish	58 (79.5%)	39 (73.6%)	39 (69.6%)	136 (74.7%)	
Arab	12 (16.4%)	12 (22.6%)	17 (30.4%)	41 (22.5%)	
Other	3 (4.1%)	2 (3.8%)	0 (0.0%)	5 (2.7%)	
Socioeconomic status ^¶^, *n* (%)					NS
Low (1–3)	6 (8.2%)	9 (17.0%)	13 (23.7%)	28 (15.5%)	
Medium (4–7)	27 (37.0%)	22 (41.5%)	17 (30.9%)	66 (36.5%)	
High (8–10)	40 (43.7%)	22 (41.5%)	25 (45.4%)	87 (48.1%)	
SDI score, mean ± SD	0.0 ± 0.0	1.41 ± 0.50	5.29 ± 2.29	1.88 ± 2.34	<0.0001
EULAR/ACR criteria cumulative score, mean ± SD	20.78 ± 9.03	21.87 ± 7.50	25.27 ± 9.01	22.46 ± 8.77	0.013
Comorbidities					
APS, *n* (%)	3 (4.1%)	15 (28.3%)	56 (44.6%)	43 (23.6%)	<0.0001
HTN, *n* (%)	8 (11.0%)	15 (28.3%)	36 (64.3%)	59 (32.4%)	<0.0001
Systolic BP last, mmHg, mean ± SD	122.9 ± 20.6	123.4 ± 16.7	134.1 ±22.3	126.9 ± 20.7	0.007
Diastolic BP last, mmHg, mean ± SD	71.9 ± 12.8	73.9 ± 10.9	78.0 ± 12.1	74.6 ± 12.2	0.031
ESRD, *n* (%)	0 (0.0%)	0 (0.0%)	9 (16.1%)	9 (4.9%)	<0.0001
DM, *n* (%)	3 (4.1%)	2 (3.8%)	13 (23.2%)	18 (9.9%)	<0.0001
CVD, *n* (%)	0 (0.0%)	6 (11.3%)	13 (23.2%)	19 (10.4%)	<0.0001
Laboratory:					
Low C3 and/or C4 ever, *n* (%)	49 (67.12%)	36 (67.92%)	41 (73.12%)	126 (69.23)	NS
Positive anti-dsDNA ever, *n* (%)	51 (69.9%)	43 (81.1%)	44 (78.6%)	138 (75.8%)	NS
aPL antibodies, ever, *n* (%)	27 (37.0%)	28 (52.8%)	27 (48.2%)	82 (45.1%)	NS
Lymph. min., K/microL, mean ± SD	1.18 ± 0.71	1.03 ± 0.68	0.74 ±0.61	0.99 ± 0.69	0.003
HGB min., g/dL, mean ± SD	11.39 ± 1.64	10.70 ±2.24	9.22 ± 2.08	10.45 ± 2.18	<0.0001
PLT min., K/microL, mean ± SD	186.00 ± 73.53	172.87 ± 60.05	133.75 ± 65.63	164.38 ± 70.43	<0.0001
eGFR, last, ml/min, mean ± SD	115.94 ± 35.01	99.27 ± 34.33	64.43 ± 45.39	93.53 ± 44.30	<0.0001
Medications, *n* (%):					
HCQ, *n* (%)	57 (78.1%)	43 (81.1%)	31 (55.4%)	131 (72.0%)	NS
GCS, ever, *n* (%)	49 (67.1%)	44 (83.0%)	51 (91.1%)	144 (79.1%)	0.001
GCS dosage *, mean ± SD	1.73 ± 3.33	2.36 ± 3.69	4.43 ± 5.66	2.75 ± 4.40	0.002
Immunosuppression, ever, *n* (%)	19 (26.0%)	17 (32.1%)	20 (35.7%)	56 (30.8%)	0.005
Cyclophosphamide, ever, *n* (%)	7 (9.6%)	11 (20.8%)	14 (25.0%)	32 (17.6%)	<0.0001
MMF, ever, *n* (%)	19 (26.0%)	16 (30.2%)	19 (33.9%)	54 (29.7%)	0.005
Azathioprine, ever, *n* (%)	16 (21.9%)	17 (32.1%)	20 (35.7%)	53 (29.1%)	<0.0001
Belimumab, ever, *n* (%)	14 (19.2%)	14 (26.4%)	13 (23.2%)	41 (22.5%)	NS
Outcomes					
Mortality, *n* (%)	0 (0.0%)	1 (1.9%)	9 (16.1%)	10 (5.5%)	<0.0001
ED visits per patient, mean ± SD	3.22 ± 5.06	4.46 ± 5.10	8.35 ± 9.13	5.16 ± 6.89	<0.0001
Hospitalizations: SLE exacerbation, *n* (%)	13 (19.7%)	11 (22.9%)	24 (46.2%)	48 (28.9%)	0.004
Severe infection, *n* (%)	8 (12.1%)	11 (22.9%)	27 (51.9%)	46 (27.7%)	<0.0001
CVE, *n* (%)	1 (1.5%)	11 (22.9%)	15 (28.8%)	27 (16.3%)	<0.0001

All variables measured at last follow-up unless stated otherwise. SDI: Systemic Lupus International Collaborating Clinics/American College of Rheumatology Damage Index; EULAR: European Alliance of Associations for Rheumatology; ACR: American College of Rheumatology; APS: antiphospholipid syndrome; HTN: hypertension; BP: blood pressure; ESRD: end-stage renal disease; DM: diabetes mellitus; CVD: cardiovascular disease including stroke, congestive heart failure and ischemic heart disease; aPL: antiphospholipid antibodies including anticardiolipin antibodies or anti-beta2glycoprotein 1 antibodies or lupus anticoagulant; lymph.: lymphocyte count; HGB: hemoglobin; PLT: platelets; min.: minimum value; eGFR: estimated glomerular filtration rate; HCQ: hydroxychloroquine; GCS: glucocorticosteroids; MMF: mycophenolate mofetil or mycophenolic acid; ED: emergency department; SLE: systemic lupus erythematosus; CVE: cardiovascular event. ^¶^ Socioeconomic status was defined according to the municipal socio-economic index by the Central Bureau of Statistics. * Corticosteroids dosage of prednisone or prednisone equivalent.

**Table 2:** Multivariable analysis for predictors of damage.

	Odds Ratio	95% CI		*p* Value
		Lower	Upper	
Sex	0.512	0.188	1.391	0.189
Age at diagnosis	1.004	0.977	1.032	0.787
Ethnicity	1.351	0.547	3.337	0.515
Hypertension	6.460	2.544	16.399	<0.001
Antiphospholipid syndrome	15.704	4.260	57.891	<0.001
Depression	1.734	0.417	7.216	0.449
Anxiety	1.191	0.312	4.543	0.798

## Data Availability

The data presented in this study are available from the corresponding author upon reasonable request.
